# Astrocytosis, Inflammation, Axonal Damage and Myelin Impairment in the Internal Capsule following Striatal Ischemic Injury

**DOI:** 10.3390/cells12030457

**Published:** 2023-01-31

**Authors:** Marco Aurelio M. Freire, Rafael Rodrigues Lima, Leonardo Oliveira Bittencourt, Joanilson S. Guimaraes, Daniel Falcao, Walace Gomes-Leal

**Affiliations:** 1 Graduate Program in Health and Society, University of the State of Rio Grande do Norte (UERN), Mossoró 59610-110, Brazil; 2 Laboratory of Functional and Structural Biology, Institute of Biological Sciences, Federal University of Pará (UFPA), Belém 66075-110, Brazil; 3 Laboratory of Experimental Neuroprotection and Neuroregeneration, Institute of Collective Health, Federal University of Western Pará (UFOPA), Santarém 68040-470, Brazil; 4 VCU Health Systems, Virginia Commonwealth University (VCU), Richmond, VA 23284, USA

**Keywords:** stroke, secondary degeneration, inflammation, internal capsule, rat

## Abstract

Secondary degeneration is defined as a set of destructive events that damage cells and structures that were initially spared or only peripherally affected by the primary insult, constituting a key factor for functional impairment after traumatic brain injury or stroke. In the present study, we evaluated the patterns of astrocytosis, inflammatory response, axonal damage and oligodendrocytes/myelin impairment in the internal capsule following a focal injection of endothelin-1 (ET-1) into the dorsal striatum. Animals were perfused at 1, 3 and 7 post-lesion days (PLD), and tissue was processed to immunohistochemistry for neutrophils (MBS1), macrophages/microglia (ED1), astrocytes (GFAP), axonal lesion (βAPP), oligodendrocytes (Tau) and myelin (MBP). A significant number of neutrophils was observed at 1PLD, followed by intense recruitment/activation of macrophages/microglia at 3PLD and astrocytic reaction with a peak at 7PLD. Oligodendrocyte damage was pronounced at 3PLD, remaining at 7PLD. Progressive myelin impairment was observed, with reduction of immunoreactivity at 7PLD. Axonal lesion was also identified, mainly at 7PLD. Our results indicate that acute inflammatory response elicited by the ischemic insult in the striatum can be associated with the axonal impairment and damage of both oligodendrocytes and myelin sheath identified in the internal capsule, which may be related to loss of tissue functionality observed in secondary degeneration.

## 1. Introduction

According to the Global Burden of Disease (2019), stroke ranks second among the leading causes of both long-term disabilities and deaths globally [1], occasionally causing physical and cognitive impairments, [2,3] resulting in a significant socioeconomic burden [4,5]. Overall, high blood pressure, age, obesity, sedentary lifestyle, drinking and smoking are listed among the main risk factors contributing to the onset of a stroke [6,7,8,9].

Stroke, according to the classical definition provided by the World Health Organization in the 1970s and still adopted, is “a focal (or at times global) neurological impairment of sudden onset, and lasting more than 24 h (or leading to death) and of presumed vascular origin” [10], being classified into hemorrhagic or ischemic [11]. The first affects 15% of stroke sufferers and is caused by a rupture of a blood vessel, resulting in bleeding directly over the brain tissue [12], responsible for majority of deaths. The second accounts for the remaining 85% of all cases [12], arising from the blockage of a blood vessel by a clot, resulting in the disruption of blood flow to underlying areas of the neural parenchyma, with subsequent compromised supply of oxygen and nutrients to the surrounding regions, leading to a breakdown of tissue physiology [11].

The complexity underlying the pathophysiology of stroke remains to be comprehensively elucidated. Critical events associated with disturbances in the cellular milieu, such as inflammatory response, ionic imbalance, oxidative stress and excitotoxicity, which ultimately result in cell death, are widely recognized as having a pivotal role in the events underlying ischemic stroke [13,14,15].

Secondary degeneration is defined as a set of destructive events that damage cells and structures that were initially spared or only peripherally affected by the primary insult, constituting a key factor for functional impairment after traumatic brain injury or stroke by oxidative stress, excitotoxicity and neuroinflammation [16,17,18]. Excitotoxicity is defined as an excessive activation of glutamate receptors by excitatory amino acids, triggering the production of free radicals and oxidative stress as a result of a dysregulation of calcium homeostasis in the brain parenchyma, ultimately resulting in cell death [16]. Oxidative stress results from an imbalance between the oxidative and antioxidant systems of cells, resulting in excessive production of free radicals and reactive oxygen species (ROS) [19]. Neuroinflammation, in turn, is a natural defense mechanism against a variety of pathologic insults in the nervous system, acting to contain and repair the local tissue damage. Nevertheless, an excessive inflammatory response can be detrimental, eventually exacerbating the tissue damage [14,19].

Animal models are essential for a comprehensive characterization of the pathophysiological events associated with stroke, mostly concerning secondary injury. In line with this, our group has published a set of studies using endothelin-1 (ET-1) as a model of stroke [20,21,22,23], in order to evaluate the patterns of the inflammatory response, demyelination and cell death in distinct regions of the nervous system. In the present study, we sought to expand this analysis, by evaluating the secondary lesion in the internal capsule, a subcortical structure constituted of bundles of white matter myelinated ascending and descending motor and sensory projections fibers located contiguously to the striatum, after acute focal injection of ET-1 into the dorsal striatum 1, 3 and 7 post-lesion days (PLD).

## 2. Materials and Methods

### 2.1. Ethical Statement and Experimental Groups

**Figure 1:**
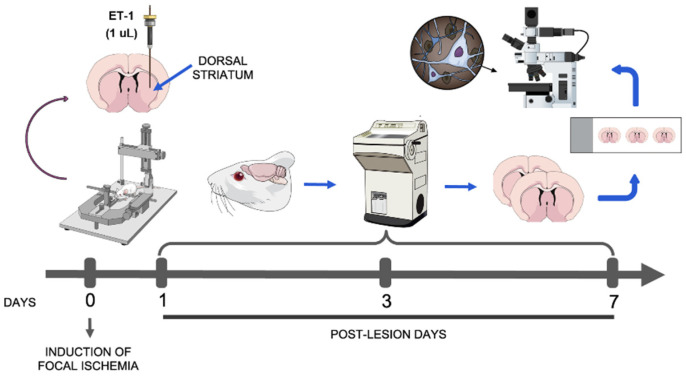
Schematic figure summarizing the methodological design. The animals were placed in a stereotaxic apparatus and a focal ischemia was induced by injection of endothelin-1 (ET-1) into the dorsal striatum. After the specified post-lesion times, animals were perfused and their brains sectioned and processed for being evaluated under bright-field microscopy.

Twenty male adult Wistar rats (280 ± 25 g) obtained from the Animal Facility at the Federal University of Pará (UFPA) were used. During the experimental period, the animals were housed in a climate-controlled room (25 ± 2 °C) with dark–light cycle of 12 h (lights on at 07:00 a.m.) in individual cages, with free access to water and food. All experimental procedures were carried out under license from the Local Ethics Committee on Experimental Animal Research (#BIO-038-12) in accordance with the NIH Guide for the Care and Use of Laboratory Animals (NIH Publications No. 80-23, revised 1985). All efforts were made to reduce the number of animals used and to avoid their distress and discomfort. Figure 1 summarizes all methodological procedures adopted.

### 2.2. Induction of Striatal Focal Ischemia

The ischemic stroke protocol was previously described by our group [20,22]. Five rats were randomly assigned for each experimental group: control, 1, 3 and 7 post-lesion days (PLD). Animals were anesthetized with a mixture of ketamine hydrochloride (Ketalar, Parker-Davis, Detroit, MI, USA) and xylazine hydrochloride (Rompun, Bayer, Leverkusen, Germany) (1.8 mg/kg and 0.5 mg/kg, respectively) (i.p.), and, after abolishment of their corneal reflex, were placed in a stereotaxic apparatus (David Kopf Instruments, Tujunga, CA, USA). A homoeothermic blanket unit was used to maintain animal’s body temperature, as measured by a rectal thermometer, which allowed evaluation of any body temperature variation among the groups. Afterward, a small craniotomy was made, followed by the injection of 1 μL of endothelin-1 (ET-1; 80 nmol/μL in sterile saline solution; Sigma Company, St Louis, MO, USA) into the left dorsal striatum over a period of 2 min, using a Hamilton syringe (Hamilton Company, Reno, NV, USA) following the stereotaxic coordinates (in millimeters relative to bregma): −1.0 mm anteroposterior (AP); 3.5 mm mediolateral (ML); and 4.5 mm dorsoventral (DV) [24]. The syringe was left stationary for 2 min to avoid reflux of the solution, being slowly withdrawn. Control animals were injected with 1 μL of vehicle (sterile 0.9% saline solution) following the same approach. At the end of the surgical procedures, all animals were returned to their individual cages and monitored daily. By adopting this experimental design, we avoided using additional animals, in accordance with the 3R’s principle of animal research [25], since that the control group is used to assure tissue damages caused by ET-1 microinjections instead a mechanical damage induced by the injection itself.

### 2.3. Perfusion and Tissue Processing

After the specified survival times (PLDs 1, 3 and 7; *n* = 5 per PLD), animals were deeply anesthetized with sodium pentobarbital (90 mg/kg; Abbott Laboratories, Abbott Park, IL, USA) (i.p.). After the abolishment of both corneal and paw withdraw reflexes, animals were perfused transcardially with successive volumes of cold 0.9% saline solution, heparinized (Roche Pharmaceuticals, Nutley, NJ, USA; 2 mL/1000 mL) and 4% cold paraformaldehyde in 0.1M phosphate buffer (PB), pH 7.4 [26]. The brains were then removed from the skulls, cryoprotected in 30% buffered-sucrose solution for 24 h, frozen in Tissue Tek (Sakura Finetek, Tokyo, Japan), and sectioned using a cryostat (Carl Zeiss/Micron, Jena, Germany). Sagittal sections of 30 µm were mounted on silanized glasses (StarFrost, Waldemar Knittel Glasbearbeitungs GmbH, Braunschweig, Germany) and stored in a freezer at −20 °C for upcoming histological analysis.

#### 2.3.1. Immunohistochemical Protocol

To perform immunohistochemistry, we adopted the protocol previously published [22]. In brief, the slides were removed from the freezer and washed in 0.1M phosphate buffer saline (PBS) for 5 min at room temperature (~25 °C). Sections were then submitted to a pretreatment in 0.2M boric acid (pH 9.0) previously heated to 65 °C for 25 min, in order to improve labeling intensity. The slides were then allowed to cool down in this solution for 20 min and were incubated in 1% hydrogen peroxide in methanol under light agitation for 20 min. Slides were rinsed three times (5 min each) in 0.05% PBS/Tween (Sigma Company, St. Louis, MO, USA) and incubated with normal serum in 0.1M PBS for 1 h (Table 1). Without further rinsing, sections were incubated with the primary antibody (Table 1) diluted in 0.1M PBS for 2 h, rinsed in PBS/Tween solution (three times, 5 min each), and incubated with secondary antibody (Table 1) for 2 h, at room temperature (~25 °C). To certify the specificity of the immunohistochemical labeling, the primary antibodies were replaced by 0.1M PBS in some randomly selected sections. Next, sections were rinsed (three times, 5 min each) in 0.1M PBS and incubated in avidin-biotin-peroxidase complex (Vectastain Standard ABC kit, Vector Laboratories Inc., Burlingame, CA, USA) for 2 h. Sections were then incubated in a solution containing 0.03% 3,3′-diaminobenzidine-tetrahydrochloride (DAB) (Sigma Company, St. Louis, MO, USA) and 0.001% hydrogen peroxide in 0.1M PB for the chromogen revelation [27]. Following the DAB reaction, sections were washed in 0.1M PB (three times, 3 min each), dehydrated in a series of graded alcohols, cleared in xylol, and coverslipped with Entellan (Merck, Darmstadt, Germany) [28]. Some sections were stained with Nissl for a basic histological analysis.

#### 2.3.2. Qualitative and Quantitative Analysis

Qualitative analysis was performed in a Nikon Eclipse 50i microscope (Tokyo, Japan). Illustrative images from all experimental groups were obtained using a digital camera (Nikon DS-Ri2, Tokyo, Japan) attached to the referred microscope.

For the quantitative analysis, we evaluated the sections containing the internal capsule adjacent to the ET-1/saline-injected striatum in order to assess the number of neutrophils (MBS1^+^ cells/mm^2^), macrophages/microglia (ED-1^+^ cells/mm^2^) and oligodendrocytes (Tau-1^+^ cells/mm^2^). Cell counting was performed using a 1 mm^2^ square grid (40× objective) attached to the eyepiece of the Nikon Eclipse 50i microscope. Six counting fields located across the internal capsule per section were evaluated, throughout three sections/animal (n = 5 animals per PLD group).

Intensities of immunolabeling of APP (axonal) and MBP (myelin) were estimated by densitometric analysis using the ImageJ software, version 1.53 (http://rsb.info.nih.gov/ij/), accessed in 1 May 2022 [29]. A 0.25 mm^2^ square window was set in the ImageJ and positioned across the internal capsule (three samples per section, two sections by animal). A normalized scale based on the non-reactive cortical white matter located above the dorsal striatum was adopted to minimize the effects of within-group variability. For each animal, the average optical density (OD) was named Ctx, the white matter Wm and a contrast index C was measured according to the equation: C = (Ctx − Wm)/(Ctx + Wm).

### 2.4. Statistical Analysis

For the statistical comparison among groups, the normality of results was tested by the Shapiro–Wilk method. Average values for all measurements were then compared among the groups using one-way ANOVA, following by Tukey post hoc test with significance level set at 95% (*p* < 0.05) using GraphPad Prism software (version 5.0, GraphPad Software Inc., San Diego, CA, USA). The results were expressed as mean ± standard error of the mean (SEM). In order to minimize any bias during quantification, only one researcher performed the analyses. The data obtained were then evaluated independently by two other researchers, both of them unaware of the data’s origin. Next, all analyses were compared to seek for variations.

## 3. Results

### 3.1. Basic Histology Reveals Cell Death Induced by ET-1 in the Striatum

**Figure 2:**
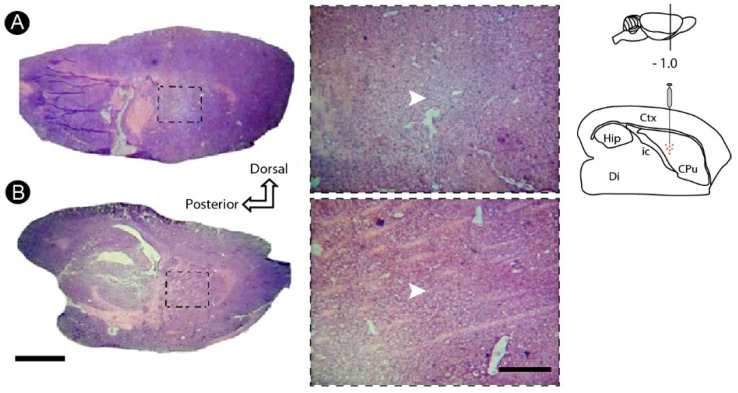
Basic histology (Nissl staining) following striatal endothelin-1 (ET-1) injection. There was a loss of tissue after seven post-lesion days (7PLD) (**A**), identified by a well-defined region of pallor, not observed in the control animal (**B**). Dashed squares in lower magnifications point to the location where higher power was obtained. Arrowheads point to the center of the injection. Drawings at the left side of the figure show the anatomical location of the ET-1 injection site into the striatum. Ctx: cortex; CPu: caudate putamen (striatum); Di: diencephalon; Hip: hippocampus; ic: internal capsule. Scale bars: 2 mm (lower magnification); 200 μm (enlargements).

Microinjections of ET-1 in the dorsal striatum induced a pallor at the injection site with conspicuous loss of cell bodies, revealed by the basic histology (Nissl stain) (Figure 2A), which did occur at the control group (Figure 2B).

### 3.2. Inflammatory Response and Glial Activation in the Internal Capsule Induced by ET-1

**Figure 3:**
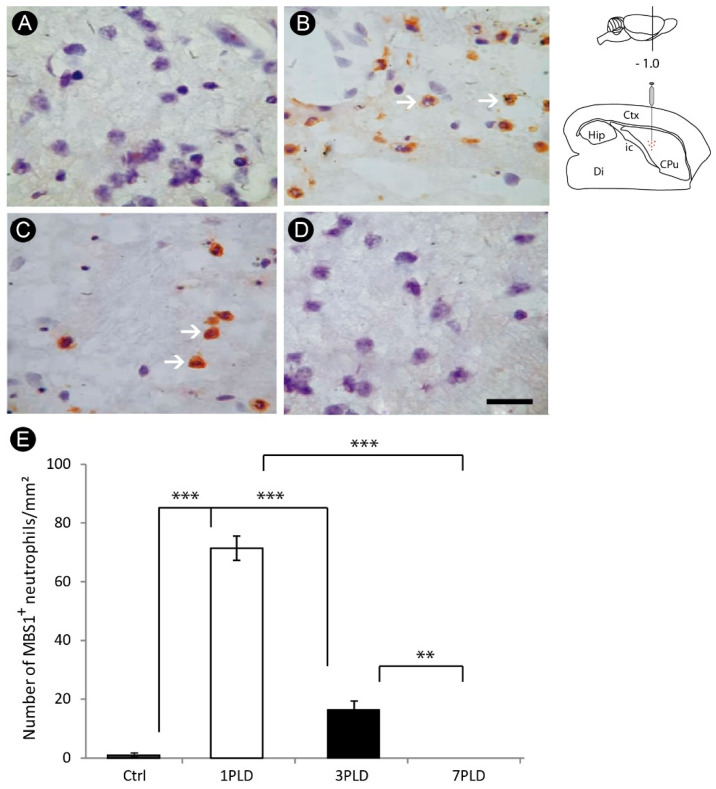
Neutrophil recruitment in the internal capsule following endothelin-1 (ET-1) injection. While in control group the inflammatory response is negligible (**A**), an intense recruitment of neutrophils is observed one day after injection (1PLD) (**B**), with a significant decrease at 3PLD (**C**). Neutrophil response is absent at 7PLD (**D**), as confirmed by quantitative analysis (**E**) (** *p* < 0.01; *** *p* < 0.001, ANOVA, Tukey post hoc test). Values expressed as mean ± SEM. Arrows in A and B point to MBS1^+^ neutrophils. Drawings at the left side of the figure show the anatomical localization of the ET-1 injection in the striatum. Ctx: cortex; CPu: caudate putamen (striatum); Di: diencephalon; Hip: hippocampus; ic: internal capsule. Scale bar: 50 µm.

An intense initial inflammatory response, evidenced by a pronounced recruitment of neutrophils, was observed in the internal capsule at 1PLD (71.4 ± 4.16 cells/mm^2^), which decreased significantly in 3PLD (16.4 ± 2.07 cells/mm^2^). This inflammatory response was not present in the later time point evaluated (7PLD) (0.0 ± 0.00 cells/mm^2^), with a negligible inflammatory response in the control group (1.1 ± 0.47 cells/mm^2^) (Figure 3A–E).

**Figure 4:**
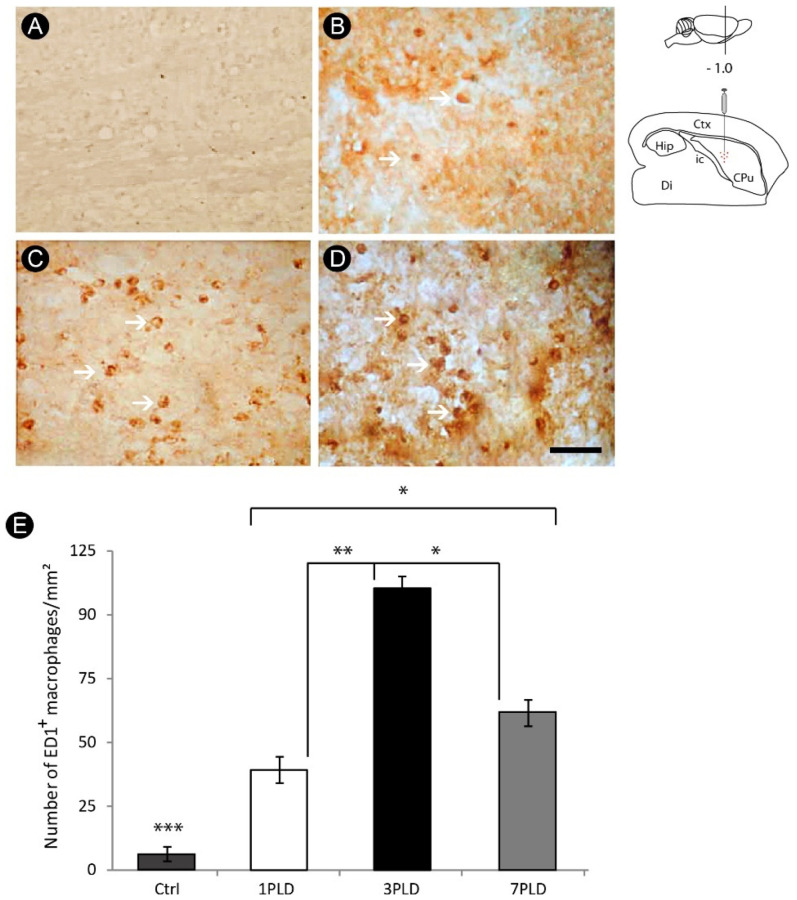
Progressive macrophage/microglia activation in the internal capsule following endothelin-1 (ET-1) striatal injection. The inflammatory response is minimal in the control group (**A**). At 1PLD it is possible to notice a small number of ED1^+^ cells (**B**), followed by a significant increase at 3PLD (**C**), with a decrease at 7PLD (**D**), as confirmed by quantitative analysis (**E**) (* *p* < 0.05; ** *p* < 0.01; *** *p* < 0.001, ANOVA, Tukey post hoc test). Values expressed as mean ± SEM. Arrows in B, C and D point to ED1^+^ cells. Drawings at the left side of the figure show the anatomical localization of the ET-1 injection in the striatum. Ctx: cortex; CPu: caudate putamen (striatum); Di: diencephalon; Hip: hippocampus; ic: internal capsule. Scale bar: 50 µm.

Nevertheless, the peak of macrophage/microglial activation occurred at 3PLD (111.6 ± 3.78 cells/mm^2^), remaining relatively high at 7PLD (60.2 ± 5.54 cells/mm^2^). The differences were statistically significant compared to control (6.8 ± 2.39 cells/mm^2^) and 1PLD groups (37.2 ± 5.54 cells/mm^2^) (Figure 4A–E).

**Figure 5:**
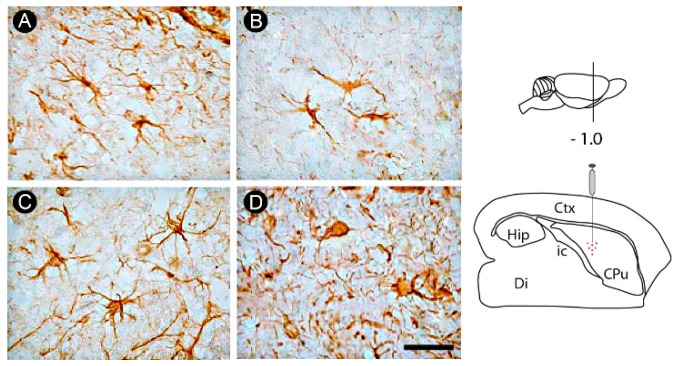
Astrocytosis in the internal capsule following endothelin-1 (ET-1) striatal injection. Non-activated astrocytes are observed in control group (**A**). A progressive astrocytosis is seen at 1PLD (**B**) and 3PLD (**C**), with hypertrophic cell bodies and short and thick processes being observed at 7PLD (**D**). Drawings at the left side of the figure show the anatomical localization of the ET-1 injection in the striatum. Ctx: cortex; CPu: caudate putamen (striatum); Di: diencephalon; Hip: hippocampus; ic: internal capsule. Scale bar: 50 µm.

A progressive glial activation was observed over the evaluated survival times in the internal capsule, characterized by alteration in the astrocyte morphology, evidenced by swelling of the cell body and shortening and thickening of processes, especially in the later time point evaluated (7PLD). In control animals, astrocytes did not present hypertrophic cell bodies and short and thick processes) (Figure 5A–D).

### 3.3. Oligodendrocyte Degeneration in the Internal Capsule Induced by ET-1 Injection

**Figure 6:**
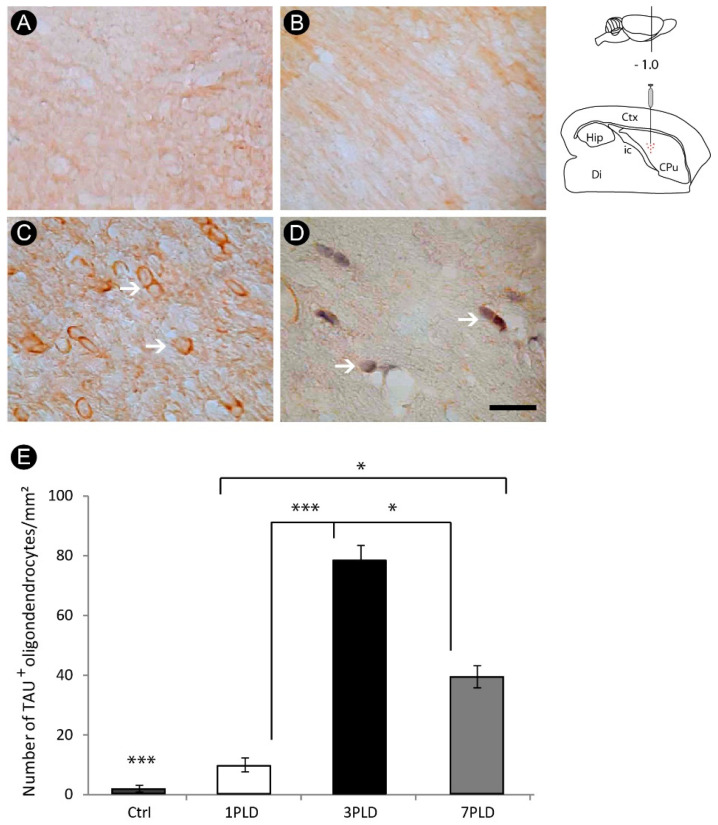
Oligodendrocyte damage in the internal capsule following endothelin-1 (ET-1) injection. Control (**A**) and endothelin-1-injected groups at 1PLD (**B**), 3PLD (**C**) and 7PLD (**D**). The peak of Tau^+^ cells is observed at 3PLD, as confirmed by quantitative analysis (**E**) (* *p* < 0.05; *** *p* < 0.001, ANOVA, Tukey post hoc test). Values expressed as mean ± SEM. Arrows in (**C**,**D**) point to TAU^+^ oligodendrocytes. Drawings at the left side of the figure show the anatomical localization of the ET-1 injection in the striatum. Ctx: cortex; CPu: caudate putamen (striatum); Di: diencephalon; Hip: hippocampus; ic: internal capsule. Scale bar: 50 µm.

Some events associated with the secondary injury were more intense at 3PLD. At that time point, it was possible to observe a conspicuous degeneration of oligodendrocytes, revealed by immunohistochemistry for Tau-1 (79.2 ± 6.02 cells/mm^2^), a cytoskeletal protein specific of these cells, compared with control (0.6 ± 0.89 cells/mm^2^) and 1PLD groups (9.8 ± 2.17 cells/mm^2^). This immunoreactivity decreased at 7PLD (39.8 ± 3.56 cells/mm^2^) (Figure 6A–E).

**Figure 7:**
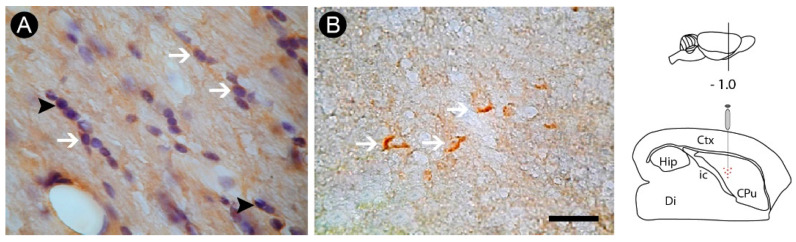
Apoptotic profiles following oligodendrocyte lesion in the internal capsule induced by endothelin-1 (ET-1) striatal injection. Nissl counterstaining revealed the presence of pycnotic bodies (arrowheads), a result confirmed by immunohistochemistry for caspase-3 (arrows) (**B**). Arrows in (**A**) point to Tau^+^ oligodendrocytes. Drawings at the left side of the figure show the anatomical localization of the ET-1 injection in the striatum. Ctx: cortex; CPu: caudate putamen (striatum); Di: diencephalon; Hip: hippocampus; ic: internal capsule. Scale bar: 100 µm.

In addition, observing Nissl counterstaining in some sections, it was possible to find evidence of apoptotic profiles in this cell group by the presence of pycnotic bodies, one of the hallmarks of apoptotic cell death (Figure 7A). This result was confirmed by immunohistochemistry for caspase-3, specific marker for apoptosis (Figure 7B).

### 3.4. Axonal Lesion in the Internal Capsule Following ET-1 Injection

**Figure 8:**
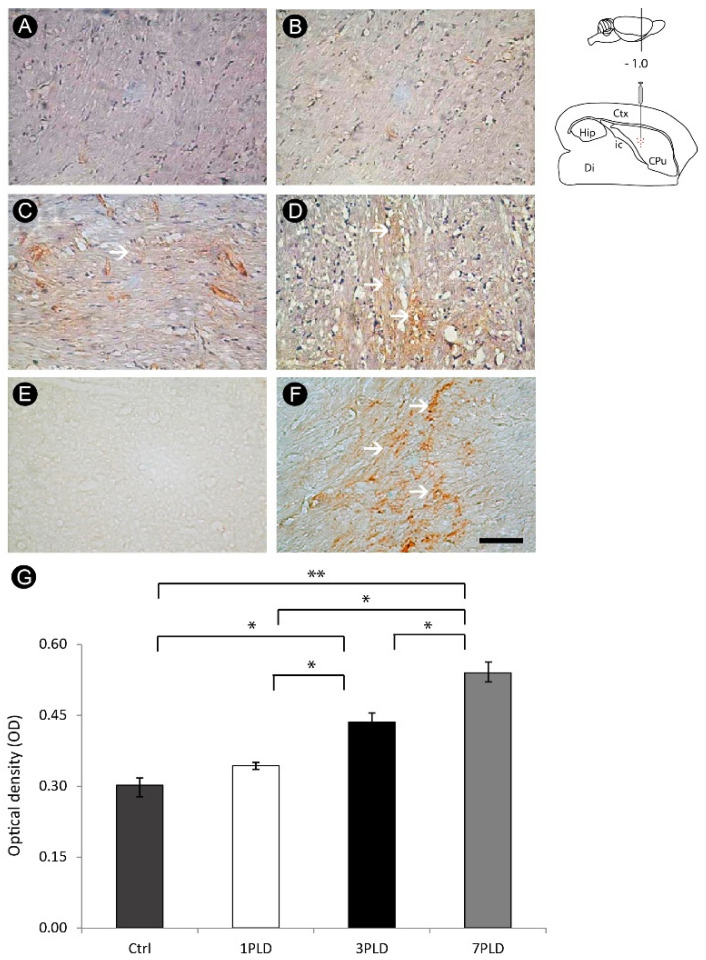
Axonal lesion in the internal capsule following endothelin-1 (ET-1) striatal injection. Control (**A**) and ET-1-injected groups at 1PLD (**B**), 3PLD (**C**) and 7PLD (**D**). The peak of APP labeling is observed at 7PLD, evidenced by a diffuse axon labeling (arrows in (**D**)). Caspase-3 immunohistochemistry allowing identifying a similar pattern of immunoreactivity, with intense labeling at 7PLD (**F**) (arrows). Control group presenting the absence of caspase-3 labeling (**E**). Progressive APP labeling across time points was confirmed by densitometric analysis (**G**) (* *p* < 0.05; ** *p* < 0.01, ANOVA, Tukey post hoc test). Drawings at the left side of the figure show the anatomical localization of the ET-1 injection in the striatum. Ctx: cortex; CPu: caudate putamen (striatum); Di: diencephalon; Hip: hippocampus; ic: internal capsule. Scale bar: 50 µm.

There was axonal damage as revealed by immunohistochemistry for APP between 1PLD and 7PLD, with peak at 7PLD. Axonal swellings were observed along the internal capsule, a pattern not observed in the control group (Figure 8A–D). Caspase-3 immunohistochemistry was also observed, with intense labeling at 7PLD, raising the possibility that axons can degenerate by apoptotic mechanism (Figure 8E–F). Densitometric quantitative analysis confirmed the qualitative data, with a progressive increase of immunoreactivity in both 3PLD and 7PLD, with peak at 7PLD, as compared with both control and 1PLD groups. There was no significant difference between control group and 1PLD group (Figure 8G).

### 3.5. ET-1 Injection in Striatum Induced Progressive Impairment of Myelin in the Internal Capsule

**Figure 9:**
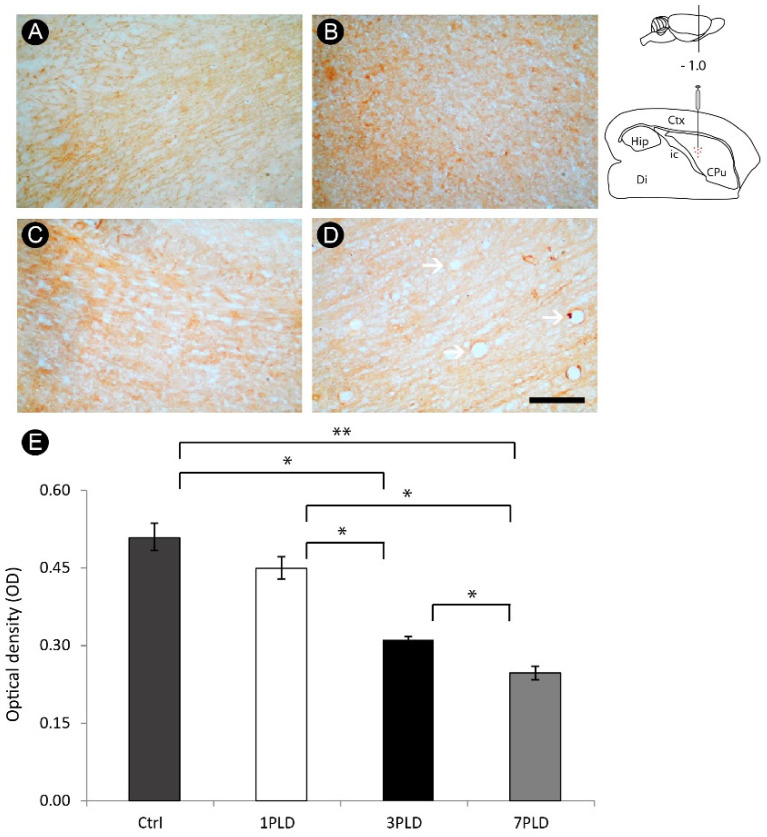
Myelin impairment in the internal capsule following endothelin-1 (ET-1) injection. Control group shows a homogeneous MBP^+^ labeling (**A**). A progressive impairment is observed at 1PLD (**B**), 3PLD (**C**) and 7PLD (**D**), with a noticeable rarefaction of reactivity in the latter survival time. Vacuolization, a hallmark of tissue degeneration, is also evident at 7PLD (arrows). Progressive loss of MBP labeling across time points was confirmed by densitometric analysis (**E**) (* *p* < 0.05; ** *p* < 0.01, ANOVA, Tukey post hoc test). Drawings at the left side of the figure show the anatomical localization of the ET-1 injection in the striatum. Ctx: cortex; CPu: caudate putamen (striatum); Di: diencephalon; Hip: hippocampus; ic: internal capsule. Scale bar: 100 µm.

There was progressive myelin degeneration throughout the time points evaluated, with a peak at 7PLD (Figure 9A–D). Considering the higher immunoreactivity for Tau-1 in earlier time points, it follows that ischemic injury induced by ET-1 primarily affects the oligodendrocyte cell body, followed by a further impairment of the myelin. In addition, vacuolization, a hallmark of tissue degeneration, is also evident at 7PLD. Qualitative analysis was further confirmed by densitometric analysis, with a progressive loss of immunoreactivity in both 3PLD and 7PLD as compared with the control group, with a peak at 7PLD. There was no significant difference between the control group and 1PLD group (Figure 9E).

## 4. Discussion

In the present study, we aimed at evaluating secondary patterns of inflammatory response, astrocytosis, axonal damage and myelin impairment in the internal capsule following a focal injection of ET-1 into the dorsal striatum. Our main results pointed an intense acute inflammatory response with neutrophils at 1PLD, followed by intense recruitment/activation of macrophages/microglia at 3PLD and astrocytic reaction with a peak at 7PLD. Oligodendrocyte damage was conspicuous between 3PLD and 7PLD. Progressive myelin impairment was observed with peak at 7PLD. Axonal lesion was also observed, mainly at 7PLD.

The internal capsule is a subcortical structure constituted by bundles of white matter myelinated ascending and descending motor and sensory projection axons located contiguously to the striatum, allowing the connection between the cerebral cortex and brainstem [30,31]. This region is potentially susceptible to cerebrovascular accidents, since the perforating arteries that supply the structure are predisposed to occlusion or rupture due to their small diameter, originating the so-called lacunar stroke [32], with minimal alterations in internal capsule resulting in severe disabilities [32,33]. In light of this, animal models evaluating the secondary damage underlying a focal stroke near the region emerge as clinically relevant.

White matter injury induced by stroke is devastating, resulting in severe disturbances associated with axonal damage, demyelination and oligodendrocyte degeneration (see [34] for review). Here, we induced a focal ischemic lesion in the dorsal striatum, not initially affecting the internal capsule. Our results are in accordance with our previous descriptions using the same model of brain ischemia, with white matter tracts being secondarily affected following ET-1 injection in regions far from the corpus callosum [23].

In the later time points evaluated, it was possible to observe significant changes in the structure of the internal capsule, associated with the inflammatory response elicited by ET-1 injection into the striatum. Primary lesion caused by both stroke and traumatic lesion initially results in the loss of tissue in the region directly affected, followed by a myriad of secondary events which ultimately affect areas spared or only marginally affected by the primary insult, resulting in functional deficits [23,35,36,37]. Our results show that the primary lesion was induced in the striatum, but inflammatory response and alterations in oligodendrocytes, myelin and axons were observed in internal capsule. Inflammation is the first line of defense of the tissue, being elicited following any sudden disturbance, in order to safeguard the affected region and also promote its recovery [38]. However, chronic inflammation can be detrimental, causing morphophysiological disturbances in the nervous system [39,40].

We identified a conspicuous astrocytic response across internal capsule especially in more latter time points (7PLD). Such an event is related to alterations in the corpus callosum and spinal cord white matter following stroke and excitotoxic response, respectively [23,36]. Depending on its intensity, astrocytosis can be beneficial or harmful following acute neural disorders, since in the late phase of injury elicited by stroke or traumatic lesion reactive astrocytes form a glial scar which physically isolates the injured region, avoiding further damage in both acute and chronic animal models [41,42].

Concerning oligodendrocyte labeling, our results revealed a significant degeneration, especially in later time points using Tau-1 as a reliable marker for pathological oligodendrocytes [20,37,43,44], confirmed by caspase-3 immunolabeling. Such a pattern was similar to our recent description in the corpus callosum [23]. The presence of pathological oligodendrocytes in the internal capsule at 7PLD indicates that oligodendrocyte degeneration can be a consequence of an exacerbated inflammatory response [23,45]. This likely affects the structure of myelin sheath, since we have identified a progressive myelin impairment, especially in the later time points. The peak of myelin degeneration was observed at 7PLD, indicating that oligodendrocyte lesion precedes myelin impairment in the internal capsule in the present stroke model.

Regarding axonal damage, we observed a progressive presence of wounded axons, with the peak of APP labeling observed at 7PLD, evidenced by diffuse axon labeling which is evidence of a progressive breakdown in the axonal structure characterized by the presence of axonal swellings. In addition, caspase-3 immunohistochemistry allowed identification of a similar pattern of immunoreactivity, with intense labeling at 7PLD.

Inflammatory response seems to be associated with progressive disturbance in oligodendrocytes, myelin sheath and axonal injury, events observed in a secondary degeneration. Since excessive inflammation plays a harmful role in nervous tissue [38,42,46], future studies using inhibitors of inflammation such as minocycline or indomethacin [21,37] or natural substances with both anti-inflammatory and antioxidative properties [47,48,49,50], can provide future opportunities for neuroprotective intervention.

Concerning detrimental effects associated with stroke, edema is one of the most significant [51]. Targeting signaling mechanisms in astrocytes emerges as a viable therapeutic option to avert harmful events related to edema generated by stroke. In a comprehensive study, Kitchen et al. (2020) showed that pharmacological inhibition of these signaling events prevents the development of nervous tissue edema and promotes functional recovery in injured rats [52]. This role has been further confirmed by Sylvain et al. (2021) using photothrombotic stroke model. Interestingly, the authors have also shown a link to brain energy metabolism, as indicated by the increase of glycogen levels [53]. In light of this, targeting these molecular and signaling mechanisms of astrocytes and other glial cells, rather than only the traditional approaches, should be explored in order to provide an effective element of functional recovering following stroke [54,55].

Our findings suggest that secondary degeneration of internal capsule following primary damage into the dorsal striatum. More investigations on the functional impairment in longer survival times using physiological and behavioral analyses, as well as the use of promising anti-inflammatory neuroprotective agents, are needed and may offer the opportunity for development of new therapeutic agents for capsular infarct.

## 5. Conclusions

Our findings show that acute inflammatory response elicited by an ischemic striatal insult can be associated with secondary axonal impairment and damage to both oligodendrocytes and myelin sheath in the internal capsule, which may be related to loss of tissue functionality observed in secondary degeneration. Further studies focusing on the neuroprotection of remote brain regions initially spared by the primary insult are required in an attempt to prevent late functional disorders.

## Figures and Tables

**Table 1:** Antibodies and normal serums used.

Primary Antibody	Secondary Antibody	Normal Serum	Labeling Purpose
Anti-MBS1 (1:2000, CNS Inflammation Group, Soton, UK)	Goat anti-rabbit (1:200, Vector Labs, Newark, CA, USA)	Goat	Neutrophils
Anti-ED1 (1:500, Serotec, Kidlington, UK)	Horse anti-mouse (1:200, Vector Labs, USA)	Horse	Activated microglia/macrophages
Anti-GFAP (1:1000, DAKO, UK)	Goat anti-rabbit (1:200, Vector Labs, USA)	Goat	Astrocytes
Anti-Tau-1 (1:500, Chemicon, USA)	Horse anti-mouse (1:200, Vector Labs, USA)	Horse	Pathological oligodendrocytes
Anti-MBP (1:100, Serotec, UK)	Horse anti-mouse (1:200, Vector Labs, USA)	Horse	Myelin
Anti-βapp (1:50, Chemicon, USA)	Horse anti-mouse (1:200, Vector Labs, USA)	Horse	Damaged axons
Anti-caspase-3 (1:250, Promega, Madison, WI, USA)	Goat anti-rabbit (1:200, Vector Labs, USA)	Goat	Apoptotic profiles

## Data Availability

All data supporting the findings of this study are included within the article.
